# Blurring Borders: Innate Immunity with Adaptive Features

**DOI:** 10.1155/2007/83671

**Published:** 2007-12-04

**Authors:** K. Kvell, EL. Cooper, P. Engelmann, J. Bovari, P. Nemeth

**Affiliations:** ^1^Department of Immunology and Biotechnology, Faculty of Medicine, University of Pécs, 7624 Pécs, Hungary; ^2^Laboratory of Comparative Neuroimmunology, Department of Neurobiology, David Geffen School of Medicine at UCLA, University of California, Los Angeles, CA 90095-1763, USA

## Abstract

Adaptive immunity has often been considered the penultimate of immune capacities. That system is now being deconstructed to encompass less stringent rules that govern its initiation, actual effector activity, and ambivalent results. Expanding the repertoire of innate immunity found in all invertebrates has greatly facilitated the relaxation of convictions concerning what actually constitutes innate and adaptive immunity. Two animal models, incidentally not on the line of chordate evolution (*C. elegans* and *Drosophila*), have contributed enormously to defining homology. The characteristics of specificity and memory and whether the antigen is pathogenic or nonpathogenic reveal considerable information on homology, thus deconstructing the more fundamentalist view. Senescence, cancer, and immunosuppression often associated with mammals that possess both innate and adaptive immunity also exist in invertebrates that only possess innate immunity. Strict definitions become blurred casting skepticism on the utility of creating rigid definitions of what innate and adaptive immunity are without considering overlaps.

## 1. INTRODUCTION: WHERE INNATE AND ADAPTIVE IMMUNITY CONVERGE

All multicellular animals (invertebrates and vertebrates) manage to keep self-integrity. Any attempt to answer questions concerning immune recognition must consider the universality of receptor-mediated responses. These may designate two forms: (1) rearranging clonally distributed antigen-specific receptors that distinguish between self and nonself according to classical Burnet hypothesis; and/or (2) pattern recognition receptors introduced by Janeway [1, 2]. The ideal immune system provides rapid and efficient responses, diverse repertoire of recognition, and effector molecules as well as specific memory on an individual level. In the self and nonself discrimination theory, the recognition receptors are central to immunity. However, a recently advanced hypothesis emphasizes that alarm signals have priority and initiate immune responses. These alarm danger signals released from the body's own cells are explained by the danger model of immunity. According to this model, immune cells must “decide” what poses harm to the body among self and nonself structures [3, 4]. The two branches of vertebrate immunity (innate and adaptive) are dependent on each other. The innate immune system, responsible for the first encounter with a pathogen, can trigger adaptive immunity in case the initial response is ineffective. Both arms interact with each other, via cell-cell interactions and soluble factors maintaining a physiological steady state [5].

With this in mind, we felt compelled to clarify and extend what seems to be the blurring or masking of certain immunological characteristics of invertebrates and vertebrates [6–8]. To do this, we first define the general features of innate and adaptive immunities. Innate immunity is considered to be natural, nonspecific, nonanticipatory, and nonclonal but germ-line encoded; whereas adaptive immunity is indeed specific, anticipatory, clonal, and somatic. Then, we discuss the blurring of vertebrate and invertebrate immunological characteristics in the following sections: (1) a preface to adaptive immunity; (2) senescence, cancer, and immunosuppressive viruses; (3) invertebrate immunological memory triggered by nonpathogenic stimuli; (4) the dawn of adaptive immunity; and (5) perspectives on innate and adaptive immunity.

## 2. A PREFACE TO ADAPTIVE IMMUNITY

### 2.1. Products of eons

Ancient innate immunity-related functions like phagocytosis and cytokine production (i.e., IL-1 and TNF) were already developed 700 million years ago in sponges and higher aquatic invertebrates (i.e., starfish). These fundamental functions remained unaltered during phylogenesis. A major evolutionary step happened 500 million years ago when fish developed jaws accompanied by evolution of the gut associated immune system. This system was fundamental to providing the genetic material required for recombination and mutation to produce variability and diversity of proteins (i.e., immunoglobulins). This system also enabled the occurrence of a wide spectrum of antigen-presenting proteins like the major histocompatibility complex (MHC). These MHC molecules developed from a primordial molecule over 300 million years ago [9].

### 2.2. Interspecies borders

A genetically colorful background is generally considered to be advantageous for species in their constant adaptation to the neighboring environment. On the other hand, for a suddenly emerging costly macroscopic function like adaptive immunity, working with clonally distributed receptors, intraspecies genetic backcrosses can make survival difficult. Therefore, in such cases, interspecies borders may help the genetic solidification of evolutionarily novel characteristics. However, drawing interspecies borders is not always easy as often seen in cases of hybridogenesis with certain invertebrate arthropods or even with vertebrate fish and amphibian species [10–12].

### 2.3. Lymphocyte receptors: survival of the fittest molecule

In the case of invertebrate organisms, species survival is maintained at the population level, which is risky for individuals. Whenever a new pathogen takes its toll, the remaining individuals are spared because they are more resistant than others. Such differences are genetically encoded [13]. However, for vertebrates, the surviving strategy is quite different. Vertebrates have a more complex immune system that generates a practically indefinite pool of recognition molecules, each present as a single cell clone. From this array of cells, those that provide better adaptation to various environments are selected in a fashion quite similar to macroscopic evolution. Cells that meet the requirements in this tough selection survive and proliferate. Such selection occurs every time a new pathogen attacks a vertebrate and the winners of this quick intercellular evolution are selected and propagated quickly enough to hunt down and neutralize the pathogen in the host organism [14].

### 2.4. Aspects of immunological ecology and evolution

Ecological immunology is a young but increasing science that examines causes and consequences of changes in immune function in the context of evolution and of ecology. Millions of invertebrate species depend exclusively on using innate immunity, in contrast to the only 45 000 vertebrate species that employ an additional acquired immune system. Regardless of this major distinction, most studies of ecological immunology discuss only vertebrates. Nevertheless, insect immunity might be more specific and similar to vertebrate immunity than previously thought [15–17].

An explanation to why an anticipatory immune system employing clonally distributed receptors has not developed in invertebrates may be provided by immunological ecology. Highly developed organisms tend to be large in size. Since the size of individual cells does not show significant interspecies variances, being larger means having more cells. Adaptive immunity works with a huge number of recognition molecules distributed in a clonal pattern. Therefore, only highly developed organisms can afford to run such a costly immune system; otherwise costs would always outweigh benefits. It seems that having huge and complex communities of cells not only demands a highly effective adaptive immune system, but actually provides its basic framework in order to exist [18, 19].

## 3. SENESCENCE, CANCER, AND IMMUNOSUPPRESSIVE VIRUSES

### 3.1. Is senescence relevant to understanding immunity?

Senescence and age-related research isa promising approach that discovers revolutionary data. Immunological senescence of vertebrate adaptive immunity is a process widely accepted by most immunologists. This is, however, less evident when thinking in terms of invertebrate innate immunity. However, this will likely change in the near future as there is accumulating evidence of senescence and more specifically immunological senescence in invertebrate species.

Morphological features of the aging process (senescence) have been recognized for many years in invertebrates. For example, when earthworms are maintained for long periods in the laboratory, a progressive decrease in size reminiscent of degeneration and a kind of wasting syndrome occur [20]. Congo red staining indicates the presence of amyloid in every organ-system as a diagnostic feature of aging [21]. With invertebrates and from a comparative viewpoint, there are examples of (1) rapid senescence and sudden death (progeria); (2) gradual senescence with definite life span; (3) negligible senescence; and (4) genetic influence on life span, mortality rates, and age-related diseases [22]. Increased activation of the immune system is a general characteristic that accompanies senescence in animals, including mammals and certain invertebrates. Gene expression analyses show that some of the most remarkable transcriptional changes that happen during aging are related to immunity. As a consequence, the use of invertebrate model organisms is highly desirable.

During senescence, *Drosophila melanogaster* expresses increasing levels of numerous antimicrobial peptides if exposed to septic bacterial infections, but not in response to bacterial extracts [23]. Mortality factor on chromosome 4 (MORF4) is known to initiate senescence in a number of cell lines. MORF-related gene on chromosome 15 (MRG15 expressed from yeast to humans) has been shown to be extremely conserved. The significant effect of MRG1 (the *Caenorhabditis elegans* ortholog of the above MRG15) in the aging process has also been demonstrated [24]. The DAF family of transcription factors supports its critical importance in the control of aging (immunosenescence) in this nematode model. The DAF-2 mediated insulin signaling pathway is a key cascade that influences senescence in *Caenorhabditis elegans* and this function seems to be evolutionarily conserved: the DAF pathway also affects aging in *Drosophila melanogaster* and rodents [25]. Innate immune functions in *Caenorhabditis elegans* are also regulated by the TGF*β*-like and the p38 MAPK pathways. The requirement of the DAF-2 cascade in regulating senescence and immunity raises molecular-level linkage of these processes [26].

### 3.2. Cancer and immunosuppressive viruses in invertebrates

#### 3.2.1. Cancer development

Cancer development has often been addressed in vertebrate species especially its relation with adaptive immunity. However, invertebrates also develop tumors in response to environmental carcinogens. Studying cancer development in species possessing innate immunity alone is a very promising field of research and may highlight adaptivelike functions present in invertebrates.

Mussels are vulnerable to several environmental toxicants and carcinogens. DNA sequence alignment of the *Mytilus edulis* homologue of vertebrate *ras* and p53 demonstrates extreme evolutionary conservatism in active domains, including four mutational hot spots [27]. Cases of transmissible sarcoma caused by environmental carcinogens (i.e., chlordane) in the soft-shell clam *Mya arenaria* have also been reported [28–30].

*Drosophila* offers a unique platform for the rapid identification and characterization of tumor suppressor genes, many of which have mammalian homologues. Genomewide microarray analysis of *Drosophila* brain tumor caused by the disfunction of the *Brat* tumor suppressor gene has identified over three hundred associated genes. Sixty of these sequences show homology to existing mammalian genes involved in tumor development [31]. As in human cancers, loss of heterozygosity can lead to tumor formation as reported in the case of the warts (*wts*) sequence. The *wts* sequence was identified by the massive overgrowth of clones homozygous for *wts* deletion [32, 33]. Similarly, mutations of the fat locus cause hyperplastic overgrowth of the imaginal discs. The affected protein product is a relative of cadherins, which are known to play an important role in human tumor suppression [34].

#### 3.2.2. Immunosuppressive viruses

For those who believe in the orthodox split between innate and adaptive immunities in terms of characteristics, it is perhaps difficult to accept the existence of viruses that specifically suppress the cellular components of innate immunity. Nevertheless, as proved by experimental data, innate immunity-specific immunosuppressive viruses exist. *Cotesia congregata* is a wasp that injects its eggs into the host caterpillar *Manduca sexta*. However, in this particular host-parasite relation, the presence of a third partner is necessary for successful parasitism: a bracovirus. The *C*. *congregata* bracovirus (CcBV) is injected simultaneously with the wasp eggs. Expression of viral genes hijacks the caterpillar's immune defense responses, which favors the survival and development of adult parasitoid wasps [63, 64]. This parasitoid wasp is known to take advantage of yet another virus in a similar fashion, a polydnavirus. Polydnaviruses (PDVs) also suppress the immune system of the host and allow the juvenile parasitoids to develop without being encapsulated by host hemocytes [65]. In invertebrates, the ambivalent relation of viruses and their hosts is further complicated by presence of both specific (RNA interference-mediated) and nonspecific (interferon-mediated) antiviral responses supporting the *blurring* of immunological functions [66].

## 4. INVERTEBRATE IMMUNOLOGICALMEMORY TRIGGERED BY NONPATHOGENIC STIMULI

### 4.1. Protostomes

**Figure 1:**
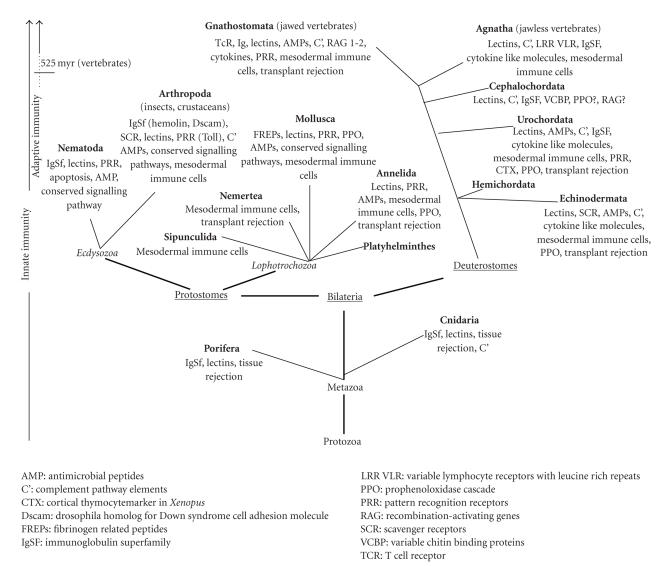
*Phylogenetic tree of the animal kingdom highlighting the evolution of key immunological elements.* Two arrows on the left side of Figure 1 indicate possible appearance of the two branches of immunity. Innate immunity may be observed along the entire animal kingdom. Traditionally accepted adaptive immunity appeared only in vertebrates, while certain adaptive immune mechanisms may have appeared early at the level of arthropods and molluscs illustrated by dots (below the arrow).

Numerous examples have been presented of animal immune responses that may develop following challenge by pathogenic organisms or nonpathogenic stimuli [8]. Here, we refer to reports previously neglected thus widening the scope of definitions of what may trigger invertebrate memory and further adaptive immunity-related features (Table 1). Most evidence concerning the evolution of innate immunity has been derived from two ecdysozoan species: *C. elegans* and *Drosophila*. In contrast, the lophotrochozoan systems share some distinct differences; mollusks may have managed immunological defense in a special manner similar to the annelids including earthworms [67] (Figure 1).

Early invertebrates present numerous examples of nonself recognition. Two classes of receptors with Ig-like domains have been identified in marine sponges: receptor tyrosine kinases and adhesion molecules. The expression of these molecules is known to be upregulated following a grafting process [35, 36, 68].

Various worm species have been used in tissue transplantation experiments. The marine nemertean ribbon worm *Lineus* readily rejects xenogeneic grafts revealing a memory component that lasts for three months [39–42]. In annelids (earthworms and leeches), accelerated rejection, weak specificity and short-term “memory” mediated by the cellular immune system have been reported [43–45, 69–74]. Molluscs are also capable of recognizing tissue alloantigens as demonstrated in the terrestrial slug *Incilaria fruhstorferi* after exchanging dorsal skin-allografts: immune cells infiltrated the grafts [46].

Recent knowledge of invertebrate innate immunity is mainly based on molecular data of dipteran insect species; however there is no recent information available about tissue allorecognition in these model organisms. However, several studies have indicated that the cockroach can respond to integumentary xenografts and effectively discriminate between self and allogeneic tissues [47, 48].

### 4.2. Deuterostomes

Sea urchins and sea stars exhibit immune responses against grafted tissues similar to those found in vertebrates [49, 50]. The responses of the urochordates *Styela plicata* and *Botryllus schlosseri* to tunic grafts confirm the existence of a sensitive histocompatibility system. Screening for genes differentially expressed during allorecognition in *Botryllus schlosseri* has identified a gene encoding a transmembrane protein showing close similarity to CD94/NKR-P1. The allorecognition of *B. schlosseri* is controlled by an ancient MHC-like system (called Fu/HC) [51, 53, 54, 75–78].

Since the complete genome of the urochordate *Ciona intestinalis* has been sequenced, it allows for the rapid identification of early evolutionary roots of adaptive immunity. In the hemocytes of *C. intestinalis,* certain adaptive-immunity homologous ESTs have been identified including vWF-like (von Willebrand factor-like), distant homologues of type I interferon (IFN) receptors, and C6-like (complement 6-like) elements [79, 80]. Moreover, genes that encode molecules with membrane receptor features of the immunoglobulin superfamily (IgSf) have also been reported [81].

## 5. THE DAWN OF ADAPTIVE IMMUNITY

The emergence of adaptive immunity was not a sudden event; its far-reaching evolutionary roots are currently under investigation by modern molecular biological methods. Genomewide sequence analysis of invertebrates has focused on the genes of innate immunity including complement components, Toll-like receptors, and those involved in intracellular signal transduction of immune responses. Assessment of extracellular C-type lectins, immunoglobulin domains, intracellular immunoreceptor tyrosine-based inhibitory motifs (ITIMs), and immunoreceptor tyrosine-based activation motifs (ITAMs) (together with their associated signal transduction molecules) suggests that activating and inhibitory receptors have an early evolutionary origin [82].

After decades of anticipation, the ancestors of some cytokines—soluble intercellular signaling molecules that form a complex network for the regulation of immunity—have recently been identified. In vertebrates, helical cytokines inlude IL2, IL6, INF *α*−1, and GM-CSF. Malagoli et al. have identified a putative helical cytokine in *Drosophila melanogaster* by elaborate bioinformatics transcriptome analysis. It is very promising that transcription from this homologue is upregulated in parallel with the known antimicobial factors defensin and cecropin A1 following Gram− or Gram+ challenge [83, 84]. Similarly, Söderhäll et al. have identified a prokineticin (PK) domain in astakine, an endogenous cytokine-like factor from the freshwater crayfish *Pacifastacus leniusculus* by mass spectrometry and PCR using degenerate primers. An astakine homologue has also been identified in the shrimp *Penaeus monodon*. In vertebrates, PK domains direct angiogenic growth. It has been demonstrated that injections of recombinant astakine actively influence differentiation and growth of hemopoietic stem cells in vivo [85].

It is a notable observation that even our most distant vertebrate relatives, jawless fish (hagfish, lamprey), have an adaptivelike immune system. It operates by means of clonally distributed leucine-rich repeat (LRR) receptors (similar to Toll-like receptors) using a novel mechanism of gene rearrangement other than RAG. These LRR modules constitute the variable lymphocyte receptors (VLRs). Computer-assisted prediction suggests a repertoire of approximately 10^14^ unique VLR receptors [86–89]. In response to the results described above, one suggestion involves the use of a different terminology for vertebrates instead of “adaptive” or “acquired” immune system: AIS or antibody-based immune system [90]. Recent studies performed in noncanonic invertebrate model-species indicate that the tracks of adaptive immunity may be much deeper than previously suggested, referring to adaptivelike immunological functions present in invertebrates [91].

## 6. PERSPECTIVES ON INNATE AND ADAPTIVE IMMUNITY

**Figure 2:**
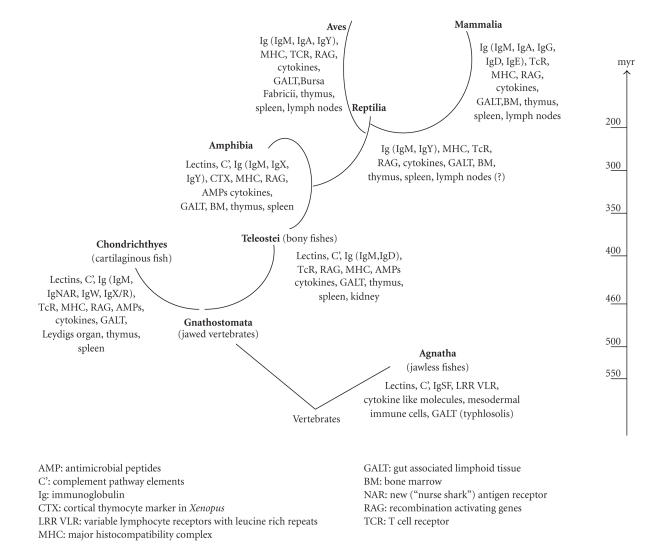
*Evolution of molecular and histological structures of the vertebrate immune system.* Regarding lymphatic tissues, the thymus, and spleen appeared early in fishes, while lymph-filtering lymph nodes are observed only in birds and mammals. Among the development of various immunoglobulin isotypes, IgD is expressed in bony fishes, later only mammals are using this B-cell receptor [55].

**Figure 3:**
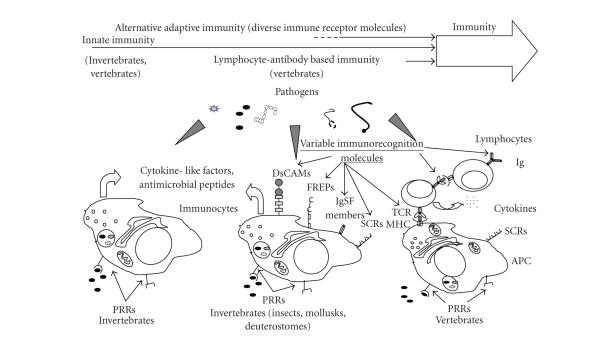
*Schematic representation of innate and adaptive immune feature development in animals.* All immune cells express nonspecific receptors, for example, pattern recognition receptors that recognize pathogen associated molecular patterns (PAMPs). Several clusters of innate receptors are conserved from plants to humans and are essential components in the defense of self-integrity. Immune cells of invertebrates also express various scavenger receptorlike proteins (Croquemort, SCRs) [37, 38, 52, 56, 57], immunglobulin superfamily members (hemolin, DsCAM) [58, 59], and fibrinogen-related peptides (FREPs) [60]; all involved in immune functions (eliminating apoptotic cells, parasites, etc.). Invertebrate immune systems also exhibit receptors with high diversity involved in immune functions: FREPs, SCRs, and DsCAMs have extreme individual variability [60–62] like vertebrate adaptive immune recognition molecules (Ig, TcR).

According to the orthodox view of phylogenetic development, immunity has reached its zenith with the emergence of the adaptive immune system (or AIS) (Figure 2). Consequently, we tend to be influenced by anthropocentric views and overlook how other highly developed organisms manage to live in hostile environments [61]. As more recent data have become available regarding nontraditional animal models, it has been suggested that the emergence of adaptive immunity is perhaps not the culmination of the evolution of immunity, but simply a successful alternative to using innate immunity alone [92]. For millions of years, many species could keep up in the continuous arms race between pathogen and host called coevolution without the surveillance of adaptive immunity [93]. The complexity of biology should never be underestimated as it turns out that those animals lacking RAG-dependent adaptive immunity can make up for an equal amount of diversity using highly variable elements of innate immunity (FREPs, DsCAM, SRCRs) finally exhibiting adaptive features [59, 92–94]. On the other hand, in vertebrates, adaptive immunity often simply serves as a sophisticated targeting device that recognizes and then processes the antigen but finally leaves the messy job of actually clearing up pathogens to the immense capacity of innate immunity. Therefore, once again we see that borders are *blurring* and the strict distinction between innate and adaptive immunities might need revision (Figure 3).

## Figures and Tables

**Table 1:** Invertebrates exhibiting induction, specificity, and/or immunological memory in the nonpathogenic context of first and second challenges with transplants (n.a.: not analyzed).

Species	Challenge	Specifity	Memory	References
**Porifera**				
*C. diffusa*	Tissue (allograft) transplantation	+	+	Smith and Hildemann,1986 [35]
*G. cydonium*		+	n.a.	Müller et al., 1999 [36]

**Cnidaria**				
*E. stricta*	Colonial contact/allograft, xenograft	+	n.a.	Theodor, 1970 [37]
*M. verrucosa*		+	+	Hildemann et al., 1977 [38]

**Nemertea**				
*L. ruber*	Tissue (allograft, xenograft) transplantation	+	+	Bierne and Langlet, 1974 [39];
*L. lacteus*				Langlet and Bierne,1975 [40]; 1982 [41]; 1984 [42]

**Annelida**				
Earthworms *L. terrestris* *E. fetida*	Tissue (allograft, xenograft) transplantation	+	+	Cooper, 1969 [43]; Cooper and Roch, 1986 [44]
Leeches *H. medicinalis* *G. complanata*	Tissue (allograft, xenograft) transplantation	+	+	Tettamanti et al., 2003 [45]

**Mollusca**				
*I. fruhstorferi*	Tissue (allograft) Transplantation	+	n.a.	Yamaguchi et al., 1999 [46]

**Arthropoda**				
*P. americana* *B. orientalis*	Tissue (allograft, xenograft) transplantation	+	+	Hartmann and Karp, 1989 [47]; Karp and Meade, 1993 [48]

**Echinodermata**				
*S. purpuratus* *L. pictus*	Tissue (allograft) transplantation	+	−	Coffaro and Hinegardner, 1977 [49]
*D. imbricata*		+	+	Karp and Hildemann, 1976 [50]

**Tunicata**				
*B. schlosseri*	Colonial contact/allograft	+	n.a.	Rinkevich et al., 1998 [51]; Scofield et al., 1982 [52];
*S. plicata*		+	+	Raftos et al., 1987 [53]; 1988 [54]
